# Incidence, Clinicopathological Features, and Outcomes of Signet Ring Colorectal Carcinoma: A Retrospective Study

**DOI:** 10.7759/cureus.74916

**Published:** 2024-12-01

**Authors:** Muhammad Zubair, Muhammad Anas Bin Akhtar, Zain Tayyab, Sayed Moosa Kazim, Aamir Syed, Shahid Khattak, Muhammad Tayyab, Hafsa Atiq, Muhammad Alauddin, Muhammad Afzaal

**Affiliations:** 1 Department of Surgical Oncology, Shaukat Khanum Memorial Cancer Hospital and Research Centre, Lahore, PAK

**Keywords:** clinicopathological features of signet ring cell carcinoma, colorectal cancer, incidence of signet ring cell carcinoma, outcomes of signet ring cell carcinoma, signet ring cell carcinoma

## Abstract

Background

Signet ring cell carcinoma (SRCC) is a rare subtype of colorectal cancer with significant variations in clinical characteristics and poor prognosis. However, there is limited data available in Pakistan. Therefore, we analyzed to examine the incidence, clinicopathological features, treatments, and outcomes of SRCC in colorectal cancer cases in Pakistan.

Methods

This study includes 214 primary signet ring cell carcinoma cases in the colon and rectum. All relevant clinical information extracts on demographic details, laboratory results, radiological findings, endoscopy, pathologic diagnoses, surgical interventions, neoadjuvant and adjuvant therapies, and their corresponding outcomes were undertaken from the same online database of hospital and analyzed using SPSS (SPSS Inc., Chicago, IL, USA), Chi-square test, and independent sample Kruskal-Wallis H test.

Results

The prevalence of SRCC was higher in younger patients (<50 years), 184 (86%). SRCC was more common in advanced stages T3 and T4, with 210 (98%) cases and 106 cases (49.5%) belonging to stage N2. In 27 cases (12.6%), there was already distant metastasis. The most common site of recurrent disease in SRCC patients was the peritoneum in about 50 (51.5%) patients, followed by multi-site metastases involving the lung, liver, bones, and lymph nodes.

Conclusion

Signet ring cell colorectal cancer (SRCC) manifests in Pakistan at a younger age and is diagnosed at a more advanced stage, often accompanied by peritoneal metastasis with elevated levels of recurrence due to relatively low rates of screening and the absence of national cancer data and guidelines. It is imperative that these issues should be addressed in order to alleviate the burden of this disease in our population.

## Introduction

Colorectal cancer (CRC) is a significant global health challenge, ranking third in incidence and second in mortality. Within Pakistan, it ranks as the fourth most common malignancy, with age-standardized (world) incidence and mortality rates of 5.4 and 3.1, respectively [1]. On the other hand, histological type is categorized in the World Health Organization classification system where non-mucinous adenocarcinoma is the most common and mucinous adenocarcinoma is the second most common. The rare kind of adenocarcinoma of the colon and rectum is signet-ring cell carcinoma (SRCC), which accounts for about 0.6-2.7% of all CRC cases. The major feature of SRCC is its high amount of intracellular mucin, which pushes the nucleus towards the edge; a cutoff point >50% signet cells is set for diagnosis [2-4].

Most variants of colorectal carcinoma exhibit similar clinical features, including bloody stools, altered bowel behavior, obstruction, and weight loss. The symptoms, however, are very late in the process: SRCC reflects an aggressive nature and advanced disease stage with a very bad prognosis. The emphasis is, therefore, on early screening, diagnosis, and timely therapeutic intervention [4-7].

This research focused on the profiling of patients with signet ring cell colorectal carcinoma using a representative institutional cohort. It has been reported that the signet ring cell variant is an unusual entity in young individuals with colorectal carcinoma in different populations, but data regarding Pakistani SRCC patients is scarce. Hence, the present study was designed to ascertain the overall frequency of signet ring cell carcinoma, clinic-pathological features, treatment, and outcomes of signet ring cell colorectal carcinoma in a subset of the Pakistani population to determine possible risk factors and screening tools for early detection and management.

## Materials and methods

Study subjects

A cohort of 2701 histologically confirmed and re-confirmed Pakistani CRC patients enrolled at Shaukat Khanum Memorial Cancer Hospital and Research Centre (SKMCH&RC) between January 2012 and December 2021. The data was collected and analyzed from the hospital’s patient database. Of these, 214 cases of SRCC in the colon and rectum were included in the current study. All relevant clinical information extracts about demographic details, laboratory results, radiological findings, endoscopy reports, pathologic diagnoses, surgical interventions, neo-adjuvant and adjuvant therapies, and their corresponding outcomes were undertaken from the same online database.

The study received approval from the Institutional Review Board (IRB) of SKMCH&RC (IRB number: EX-19-02-18-01-A1). Since it was a retrospective study using anonymous clinical data, the SKMCH IRB waived informed consent.

Operational definitions

The diagnosis of SRCC was pathologically confirmed by the presence of more than 50% signet ring cells. Colonoscopic configurations of the tumors were classified into three types: exophytic, ulcerative, and infiltrative. Exophytic means some abnormal growth projecting outward from the tissue surface. Ulcerative means the lesions are relatively depressed compared with surrounding mucosa. In contrast, infiltrative tumors had indistinct margins between the cancerous and non-cancerous tissues. Tumors were staged with the TNM staging system and the AJCC Cancer Staging Manual, 8th ed., of the American Joint Committee on Cancer, whereby patients at Stage I and II were classified as the early stages and those at Stage III and IV as advanced stages.

Patients had regular clinical follow-ups, with a three-monthly schedule for the first year, six months for the next two years, and annually afterward. Tumor progression was defined by the occurrence of either local recurrence or distant metastases, confirmed either pathologically or radiologically.

Statistical analysis

The data were analyzed using SPSS version 27.0 (SPSS Inc., Chicago, IL, USA). For categorical variables, frequencies and percentages were computed, while continuous variables were summarized by medians and interquartile ranges. Relationships between categorical variables and cancer stages were evaluated by the Chi-square test, while independent sample Kruskal-Wallis H test was used to evaluate the differences of continuous variables among various cancer stages. The results were considered statistically significant with p<0.05.

## Results

This study involved 214 primary signet ring cell carcinoma cases in the colon and rectum. Of these, 165 (77.1%) patients were male and 49 (22.9%) were female, with the average age being 37±11.8 years (range, 15-74). Of these cases, 184 (86%) were below 50 years, while 30 (14%) were above 50 years. Primary SRCC accounted for 7.9% of CRC patients. Only 42 (19.6%) subjects had systemic co-morbidities, which included diabetes mellitus in 17 (7.9%), hypertension in 19 (8.9%), ischemic heart disease in 2 (0.93%), inflammatory bowel disease in 2 (0.93%), and chronic kidney disease in 2 (0.93%). Demographic And clinical characteristics of the study cohort are given in (Table 1).

**Table 1:** Demographic characteristics

Variables	Category	All patients	Percentage %
Age	Mean±SD	33±11.8	
	<50 years	184	86
	>50 years	30	14
Gender	Male	165	77.1
	Female	49	22.9
Comorbidity	Yes	42	19.6
	Diabetes mellitus	17	7.9
	Hypertension	19	8.9
	Ischemic heart disease	2	0.93
	Inflammatory bowel disease	2	0.93
	Chronic kidney disease	2	0.93

The majority of the patients, 142 cases (66.4%), manifested with hematochezia, while others showed altered defecation patterns in 39 cases (18.2%), abdominal discomfort in 17 cases (7.9%), and obstruction in 16 cases (7.5%).

Carcinoembryonic antigen (CEA) analysis

The mean value of carcinoembryonic antigen was 18 ng/ml with a range of (0.2-326 ng/ml). It was noted that there were statistically significant high levels of CEA with advanced stages of diseases as compared to the early ones, where levels were 2.1ng/ml (3.36), 4.5ng/ml (11.26), and 5.6ng/ml (21.79) (p=0.020) during stage II, III and IV respectively (p=0.020). The average levels for hemoglobin and albumin were 12.8g/dl and 4.8g/dl respectively.

Clinicopathology

The leading location for the tumors was in the rectum or rectosigmoid junction in 152 (71%) cases, while other places were the ascending, transverse, descending, and sigmoid in 26 (12.2%), 14 (6.6%), 11 (5.1%), and 11 (5.1%), respectively. On colonoscopy, infiltrative morphology was present in most of the tumors in 154 (71.9%) cases, followed by exophytic and ulcerative morphology in 47 (21.9%) and 13 (6.2%), respectively. SRCC was more common in the advanced stage (T3, T4) 210 (98%). 106 cases (49.5%) belonged to stage N2. In 27 cases (12.6%), there was already distant metastasis. Initial staging, as per the American Joint Committee on Cancer (AJCC), categorized stages 0, 1, 2, 3, and 4 for 0 (0%), 0 (0%), 16 (7.5%), 172 (80.4%), and 26 (12.1%) patients respectively.

On histopathology, 188 cases (87.9%) were classified as poorly differentiated, and 26 cases (12.1%) as moderate to poorly differentiated. Patients in advanced stages exhibited higher positivity rates for Circumferential Resection Margin (CRM) and Extramural Vascular Invasion (EMVI) than those in early stages. Of the total 214 patients, 12 (5.6%) were inoperable due to advanced stage. Surgical procedures for the 202 (94.4%) patients are mentioned in (Table 2).

**Table 2:** Clinicopathological and oncological data of study subjects.

Variable	Category	All patients	Percentage (%)
Presentation	Bloody stool	142	66.4
	Altered bowel habits	39	18.2
	Abdominal pain	17	7.9
	Obstruction	16	7.5
Labs	Hemoglobin	12.8 g/dl	
	Albumin	4.8 g/dl	
	CEA	18 ng/ml (0.2-326)	
Colonoscopy	Yes	214	
	Infiltrative	154	71.9
	Exophytic	47	21.9
	Ulcerative	13	6.2
Tumor Location	Rectum/rectosigmoid	152	71
	Ascending colon	26	12.2
	Transverse colon	14	6.6
	Descending colon	11	5.1
	Sigmoid colon	11	5.1
T Stage	T1	1	0.5
	T2	3	1.4
	T3	162	75.7
	T4	48	22.4
N Stage	N0	28	13.1
	N1	80	37.4
	N2	106	49.5
M Stage	M0	187	87.4
	M1	27	12.6
AJCC Stage	I	0	0
	II	16	7.5
	III	172	80.4
	IV	26	12.1
Grade/differentiation	Poorly differentiated	188	87.9
	Moderate to poorly differentiated	26	12.1
	Well-differentiated	0.0	0.0
	CRM positive (radiological)	53	24
	EMVI positive (radiological)	5	2.3
Neo-adjuvant		139	65
Surgery	Yes	202	94.4
	No	12	5.6
Adjuvant		199	93
Palliative chemotherapy		99	46
Relapse		97	45

Treatment regimens

All patients with carcinoma of the rectum diagnosed to have advanced-stage disease were treated with neo-adjuvant chemotherapy, Cap OX (Capecitabine, Oxaliplatin)/Short/ long course, radiotherapy 50.4Gy/28 fractions before surgical intervention, followed by adjuvant therapy with CapOx. All patients with carcinoma of the colon were primarily treated with surgical intervention, followed by adjuvant therapy with CapOx or FolFox. Palliative chemotherapy was offered to patients with unresectable disease or recurrent disease after surgery and induction with Folfirinox, Capox, Irinotecan, and best supportive care.

The most common site of recurrent disease in this series was the peritoneum in about 50 (51.5%) patients, followed by multi-site metastases involving the lung, liver, bones, and lymph nodes, as shown in (Table 3).

**Table 3:** Surgery, site of relapse and outcomes.

Variable	Category	No of patients (N)	Percentage (%)
Surgery	Yes	202	94.4
	Right hemicolectomy	31	15.4
	Left hemicolectomy	9	4.5
	Extended right hemicolectomy	4	1.9
	Low anterior resection	49	24.3
	Abdominoperineal resection	51	25.2
	Extralevator abdominoperineal resection	41	20.3
	Hartman	6	2.9
	Pan proctocolectomy/ total colectomy	11	5.5
Site of relapse	yes	97	
	Peritoneum	50	51.5
	Liver	7	7.2
	Retroperitoneum para-aortic nodes	10	10.3
	Local/anastomotic site	5	5.15
	Bone	2	2.05
	More than one site	23	23.9
Outcome	Died of disease relapse	57	26.6
	Alive with disease relapse	35	16.3
	Treated and discharged	71	33.2
	Under treatment	26	12.2
	Lost to follow-up	25	11.7

During the five-year follow-up, 92 (42.9%) patients had disease relapse, among which 57 (26.6%) died. Of these, 35 16.3%) were alive with the disease, 71 (33.2%) were treated successfully, and the follow-up did not show any relapses and therefore were discharged. 26(12.2%) are still under treatment, and 25 (11.7%) were lost to follow-up.

## Discussion

Primary signet ring cell carcinoma SRCC is a rarely seen subtype of colorectal carcinoma CRC worldwide; however, it shows a slightly higher incidence in the East compared with the West [1]. In the cohort of 2701 persons carrying a previous diagnosis of CRC, primary SRCC involving the colon and rectum corresponded to 214 cases, accounting for 7.9% of all primary CRC malignancy cases. The incidence of Primary SRCC reported in our research cohort was higher than the frequency described in the literature by previous studies [1,8]. Studies conducted in various regions of the country have shown a rise in the prevalence of primary SRSS in Pakistan, with an increase of 5.4% reported at high-volume centers [9].

Our study found that the condition of SRCC was more prevalent in males than females. This trend replicates many findings from global research regarding the documented disparities in CRC incidence between the two sexes [10,11]. Genetic predisposition and environmental factors such as a high intake of red meat, alcoholic beverages, and tobacco smoking seem to have a more critical role in the etiology of the disease among males rather than females [10-13]. In this study, SRCC was significantly higher in younger patients (<50 years), 86%, than in older (>50 years), 14%. On the other hand, recently, a very disturbing trend associated with increased rates of CRC in the young population has been observed in the United States, Canada, and Australia [14-16]. Based on this evidence, the American Cancer Society has recently revised its recommendations for CRC screening in lower-risk individuals, which reduced the recommended age to initiate screening to 45 years [17]. A previous study showed that age ≤40 years was recognized as an independent predictor of the prognosis of the disease in patients [18]. Another research study has validated that Primary SRCC occurs four times more often in young patients under 40 years of age compared to older patients over 40 years of age [19]. This highlights the importance of conducting further research for the advancement and execution of screening initiatives.

In our research, 154 cases exhibited an infiltrative tumor configuration, accounting for 71.9% of cases. Subsequently, the exophytic type was identified in 47 cases (21.9%). These findings align with Messerini et al.'s study, which indicated that the infiltrative type dominated primary SRCC tumors at approximately 70.6%, while the exophytic type accounted for 29.4% [20]. In our study, SRCC was more prevalent in the rectum than in other colon regions. These findings may help in devising better and site-specific treatments for SRCC.

In our study, 98% of T3/T4 were identified with complaints of per-rectal bleeding, changes in bowel habits, and abdominal fullness. Laboratory workup showed high carcinoembryonic antigen (CEA) levels which is also consistent with the literature [21]. This study also reported that SRCC was diagnosed at an advanced stage T3-T4 [21]. Bonello et al. described three mechanisms for the advanced stages: a very rare tumor with mucosal spread, hardly radiologically diagnosable from an inflammatory process. By the time a patient becomes symptomatic, the tumor metastasizes through lymph-vascular invasion [22]. This study highlights the need to develop early screening strategies for a better prognosis of the disease.

Some authors advocate for extensive surgical removal with an increase in systemic chemotherapy for SRCC because of the poor prognosis [23,24]. In consideration, NCCN recommends adjuvant therapy for all patients with Stage II colon cancer and above with high-risk criteria. High-risk criteria include T4 stage, poor lymphadenectomy, poor histological differentiation, bowel obstruction or perforation, and lymphovascular or perineural invasion [25]. In patients diagnosed with the signet ring cell carcinomas of the colon, poor prognosis is attributed to advanced stages and early metastasis. Most of our patients were diagnosed at a mostly advanced stage of T3/T4, with most of them treated by surgical intervention, which reportedly proved to have a very poor prognosis compared to those with early-phase tumors.

As compared to other variants of CRC, SRCC has been reported to have significantly frequent metastases to the peritoneal surface compared to the lungs and liver [26]. In the present study, peritoneal metastasis involvement was found in 51.5%, while more than one site of involvement was noted in about 23.9% of the patients. Won-lee et al. reported that patients diagnosed with SRCC had a lowered disease-free survival rate and overall survival rate compared to patients diagnosed with adenocarcinoma. Here, the five-year follow-up mortality was noted to be 26.6% [27].

The limitations include a single-center retrospective study. There was also a lack of data concerning family history, molecular data, immunological, and targeted therapies other than chemotherapy.

## Conclusions

Signet Ring cell colorectal cancer (SRCC) manifests in Pakistan at a younger age and diagnosed at a more advanced stage, often accompanied by peritoneal metastasis with elevated levels of recurrence due to relatively low rates of screening and the absence of national cancer data and guidelines. It is imperative that these issues should be addressed in order to alleviate the burden of this disease in our population.

## References

[1] (2024). Global cancer observatory. https://gco.iarc.who.int/media/globocan/factsheets/populations/586-pakistan-fact-sheet.pdf.

[2] Nitsche U, Friess H, Agha A (2016). Prognosis of mucinous and signet-ring cell colorectal cancer in a population-based cohort. J Cancer Res Clin Oncol.

[3] Du W, Mah JT, Lee J, Sankila R, Sankaranarayanan R, Chia KS (2004). Incidence and survival of mucinous adenocarcinoma of the colorectum: a population-based study from an Asian country. Dis Colon Rectum.

[4] Chen JS, Hsieh PS, Chiang JM (2010). Clinical outcome of signet ring cell carcinoma and mucinous adenocarcinoma of the colon. Chang Gung Med J.

[5] Nitsche U, Zimmermann A, Späth C (2013). Mucinous and signet-ring cell colorectal cancers differ from classical adenocarcinomas in tumor biology and prognosis. Ann Surg.

[6] Belli S, Aytac HO, Karagulle E, Yabanoglu H, Kayaselcuk F, Yildirim S (2014). Outcomes of surgical treatment of primary signet ring cell carcinoma of the colon and rectum: 22 cases reviewed with literature. Int Surg.

[7] Hugen N, Verhoeven RH, Lemmens VE (2015). Colorectal signet-ring cell carcinoma: benefit from adjuvant chemotherapy but a poor prognostic factor. Int J Cancer.

[8] (2024). World health statistics annual Geneva: WHO databank. https://www.who.int/publications/i/item/9789240094703.

[9] Farooqui N, Khan S, Siddique S (2022). Signet ring cell colorectal cancers portend an aggressive biology: A 17-year analysis of operable colorectal cancers at a tertiary hospital in Pakistan.

[10] Cook MB, Dawsey SM, Freedman ND (2009). Sex disparities in cancer incidence by period and age. Cancer Epidemiol Biomarkers Prev.

[11] Hossain MS, Karuniawati H, Jairoun AA (2022). Colorectal cancer: a review of carcinogenesis, global epidemiology, current challenges, risk factors, preventive and treatment strategies. Cancers (Basel).

[12] White A, Ironmonger L, Steele RJ, Ormiston-Smith N, Crawford C, Seims A (2018). A review of sex-related differences in colorectal cancer incidence, screening uptake, routes to diagnosis, cancer stage and survival in the UK. BMC Cancer.

[13] Bademci R, Bollo J, Martinez MC, Hernadez MP, Targarona EM (2019). Colorectal cancer prognosis: The impact of signet ring cell. Gastrointest Tumors.

[14] Siegel RL, Fedewa SA, Anderson WF, Miller KD, Ma J, Rosenberg PS, Jemal A (2017). Colorectal cancer incidence patterns in the United States, 1974-2013. J Natl Cancer Inst.

[15] Brenner DR, Heer E, Sutherland RL, Ruan Y, Tinmouth J, Heitman SJ, Hilsden RJ (2019). National trends in colorectal cancer incidence among older and younger adults in Canada. JAMA Netw Open.

[16] Kyaw M, Sung JJ (2016). Young-onset colorectal cancer in the Asia-Pacific region. Med J Aust.

[17] Wolf AM, Fontham ET, Church TR (2018). Colorectal cancer screening for average-risk adults: 2018 guideline update from the American Cancer Society. CA Cancer J Clin.

[18] Foppa C, Maroli A, Lauricella S (2022). Different oncologic outcomes in early-onset and late-onset sporadic colorectal cancer: a regression analysis on 2073 patients. Cancers (Basel).

[19] Wang R, Wang MJ, Ping J (2015). Clinicopathological features and survival outcomes of colorectal cancer in young versus elderly: a population-based cohort study of seer 9 registries data (1988-2011). Medicine (Baltimore).

[20] Messerini L, Novelli L, Comin CE (2004). Microvessel density and clinicopathological characteristics in hepatitis C virus and hepatitis B virus related hepatocellular carcinoma. J Clin Pathol.

[21] Tawadros PS, Paquette IM, Hanly AM, Mellgren AF, Rothenberger DA, Madoff RD (2015). Adenocarcinoma of the rectum in patients under age 40 is increasing: impact of signet-ring cell histology. Dis Colon Rectum.

[22] Bonello JC, Quan SH, Sternberg SS (1980). Primary linitis plastica of the rectum. Dis Colon Rectum.

[23] Kang H, O'Connell JB, Maggard MA, Sack J, Ko CY (2005). A 10-year outcomes evaluation of mucinous and signet-ring cell carcinoma of the colon and rectum. Dis Colon Rectum.

[24] Benedix F, Kuester D, Meyer F, Lippert H (2013). [Influence of mucinous and signet-ring cell differentiation on epidemiological, histological, molecular biological features, and outcome in patients with colorectal carcinoma]. Zentralbl Chir.

[25] Benson AB, Venook AP, Al-Hawary MM (2018). NCCN guidelines insights: colon cancer, version 2.2018. J Natl Compr Canc Netw.

[26] Liang Z, Yan D, Li G, Cheng H (2018). Clinical analysis of primary colorectal signet-ring cell carcinoma. Clin Colorectal Cancer.

[27] Lee WS, Chun HK, Lee WY (2007). Treatment outcomes in patients with signet ring cell carcinoma of the colorectum. Am J Surg.

